# Editorial: Recent Discoveries in Human Serious Foodborne Pathogenic Bacteria: Resurgence, Pathogenesis, and Control Strategies

**DOI:** 10.3389/fmicb.2018.02412

**Published:** 2018-10-09

**Authors:** Lanming Chen, Walid Alali

**Affiliations:** ^1^Key Laboratory of Quality and Safety Risk Assessment for Aquatic Products on Storage and Preservation (Shanghai), China Ministry of Agriculture, College of Food Science and Technology, Shanghai Ocean University, Shanghai, China; ^2^Department of Epidemiology and Biostatistics, Faculty of Public Health, Kuwait University, Kuwait City, Kuwait

**Keywords:** foodborne bacterial pathogen, resurgence, pathogenesis, control, editorial

Billions of people in the world are at risks of unsafe food. Many millions become sick while hundreds of thousand die annually due to consumption of contaminated food (Fung et al., 2018). Outbreaks and prevalence of foodborne diseases are not only a major burden on global healthcare systems, but also result in huge negative impact on economic growth and social stability. Along with the rapid development of web-based and mobile-ready electronic commerce, the fast-paced national and international trades in foodstuffs around the globe present new challenges to food safety systems, particularly in developing nations. Pathogenic bacteria contaminate food at any stages in the entire food chain from farm to dining-table. The most commonly known bacterial pathogens associated with foodborne diseases worldwide are *Salmonella enterica, Campylobacter jejuni, Escherichia coli, Listeria monocytogenes, Cronobacter sakazakii, Vibrio cholerae*, and *Vibrio parahaemolyticus*. This Research Topic reported recent discoveries in resurgence, pathogenesis, and control strategies of these foodborne pathogens.

Continuous monitoring of food contaminants and identification of risk factors are crucial for assuring food safety. Many original research articles included in this Research Topic addressed issues related to the genetic diversity, prevalence, resistance, and novel transmission vectors of pathogenic bacteria. For example, *C. jejuni*, a leading cause of gastroenteritis in humans, can reside in food animal reservoirs such as chickens, pigs, and cattle. The genetic diversity and frequency of antimicrobial resistance of *C. jejuni* recovered from 214 cattle at three Michigan herds in USA were examined and determined in a cross-sectional study (Cha et al.). *Cronobacter* spp. can cause necrotizing enterocolitis, bacteremia, and meningitis in neonates and infants, with a 40–80% mortality rate (Holy and Forsythe, 2014). Powdered infant formula (PIF) is the most significant source of *Cronobacter* spp. resulting in the infections. The antimicrobial and desiccation resistance of 70 *Cronobacter sakazakii* and *Cronobacter malonaticus* isolates from PIF and processing environments in China were determined (Fei et al.). Non-typhoidal *Salmonella* (NTS) can infect a wide range of hosts, including humans, poultry, cattle, and other domesticated and wild animals worldwide. It is estimated that 93.8 million people experience salmonellosis annually, with nearly 155,000 deaths (Majowicz et al., 2010). The potential virulence profile, genetic relatedness, and host adaptation of avian and mammalian NTS isolates based on the bacterial antigens FimA (adhesin) and IroN (receptor) were investigated in a large number of NTS isolates of different host origins (Alshalchi et al.). The authors revealed the possible influence of non-synonymous point mutations within the FimA adhesin of the NTS isolates in the host adaptation (Alshalchi et al.). Enteroaggregative *Escherichia coli* (EAEC) is a common cause of foodborne disease. EAEC strains express a heterogeneous array of putative virulence factors; therefore, the recognition of specific pathogenic factors remains challenging. Jønsson et al. monitored a collection of 162 clinical Danish EAEC strains and identified a novel pAA virulence plasmid encoding toxins and two distinct variants of the fimbriae of EAEC. *Enterococcus faecalis* is frequently detected in mineral and spring water and can cause human urinary tract infections, endocarditis, and neonatal sepsis. The prevalence, potential virulence genes, antimicrobial resistance, and genetic diversity of *E. faecalis* isolates from 314 mineral water and spring water samples surveyed in China were also investigated (Wei et al.). Collectively, these studies highlighted a high prevalence of antimicrobial or desiccation resistant pathogenic bacteria associated with diverse genotypes.

Decades of research has identified a number of virulence determinants produced and secreted by pathogenic bacteria (Martinović et al., 2016). To establish infections in humans, pathogens must sense and respond to newly encountered host environments to regulate the expression of critical virulence factors that allow for niche adaptation and successful colonization (Bäumler and Sperandio, 2016; Lustri et al., 2017). In this Research Topic, the recently described molecular mechanisms underlying the outcompeting resident microbiota within the gastrointestinal tract by non-typhoidal serovars of *S. enterica* was extensively reviewed by Anderson and Kendall. The bacterium, such as serovar *Typhimurium* (S. Tm) directly eliminates close competitors via bacterial cell-to-cell contact as well as by stimulating a host immune response to eliminate specific members of the microbiota. Additionally, S. Tm tightly regulates the expression of key virulence factors that enable S. Tm to withstand host immune defenses within macrophages. In this context, the authors also highlighted chemical and physical signals that S. Tm senses as cues to adapt to each of these host environments (Anderson and Kendall).

Some other original articles included in this Research Topic also reported new findings in bacterial pathogenesis. For instance, the detailed functions of a cellulose biosynthesis-related gene (*bcsR*) of *C. sakazakii* was investigated using a gene knockout technique, and the results demonstrated that the *bcsR* is a negative regulator of cellulose biosynthesis but positively regulates biofilm formation and the adhesion/invasion ability of *C. sakazakii* (Gao et al.). RpoS is a key stress-inducible sigma factor that regulates stress resistance genes in *E. coli*. A novel missense point mutation at RpoS residue 128 in a clinical Shiga toxin-producing *E. coli* (STEC) isolate was identified. The hydrophobicity of the amino acid at residue 128 is critical for RpoS activity and is consequently important for bacterial survival at cold temperature and oxidative stresses (Iwase et al.). STEC strains also differ in acid resistance. When grown in minimal medium at pH 3.3, STEC strain B201 exhibiting flocculation was more acid sensitive, while STEC strain B241 was planktonic and acid resistant. Transcriptomic and targeted gene expression data showed that the expression of curli and acid induced chaperone genes *csg* and *hde* positively correlated with the phenotypic differences (Kay et al.). *Bacillus cereus* is increasingly reported to be a causative agent of human gastrointestinal disease. Jeßberger et al. investigated enterotoxin production, secretion, and cytotoxicity in a set of 19 enteropathogenic and non-pathogenic *B. cereus* strains of diverse origins by using cell culture medium pre-incubated with human colon epithelial cell line CaCo-2. The authors suggested that the currently used methods in *B. cereus* diagnostics based on standard culture medium should be complemented by cultivation procedures simulating intestinal host conditions (Jeßberger et al.).

Biological and non-biological innovation in technologies has emerged for better control of foodborne pathogens. The contemporary advances in DNA sequencing technologies have not only enabled finer characterization of bacterial genomes but also provided deeper taxonomic identification of complex microbiomes inhabiting an environment, such as a particular body econiche (e.g., human intestinal contents) and a food manufacturing facility econiche (e.g., floor drain) (reviewed by Cao et al.). In the past years, the discovery of small non-coding RNAs (sRNAs) unraveled a new world of post-transcriptional regulatory networks, which cooperate with Ribonucleases (RNases) in the control of gene expression. With the development of new technologies, many sRNA molecules were identified and shown to be important players in bacterial virulence. RNA metabolism has recently been exploited for the development of new therapeutic applications (reviewed by Matos et al.). During food processing and preservation, many foodborne pathogens can be induced to enter a viable but non-culturable (VBNC) state by limiting environmental conditions such as extreme temperatures, drying, irradiation, pulsed electric field, and high pressure stresses, as well as the addition of preservatives and disinfectants. After entering the VBNC state, foodborne pathogens cannot be detected using conventional plate counting techniques and introduce big challenges to food safety. Various features of the VBNC state was extensively reviewed by Zhao et al. including biological characteristics, induction and resuscitation factors, formation and resuscitation mechanisms, detection methods, and relationship to food safety.

New compounds and treatment strategies have been explored for the control of bacterial pathogens. For instance, Shi et al. analyzed the effects of thymoquinone, a principal active ingredient in volatile oil of Nigella sativa seeds, on the suppression of virulence-related traits of *C. sakazakii* ATCC 29544, and *in vitro* tests showed that sub-inhibitory concentrations of thymoquinone significantly decreased motility, quorum sensing, and endotoxin production of the bacterium. Furthermore, thymoquinone substantially reduced the adhesion and invasion of *C. sakazakii* ATCC 29544 to human colonic cell line HT-29 cells and decreased the number of intracellular bacterial cells within the RAW264.7 macrophage cells. *V. cholerae* can cause cholera, a severe diarrheal disease that can be quickly fatal if untreated and is typically transmitted via contaminated water and person-to-person contact (Baker-Austin et al., 2018). Outbreaks of cholera are reported every year in developing nations (World Health Organization, http://www.who.int/). The effects of dietary minerals zinc, selenium, and manganese on virulence attributes of *V. cholerae* was studied (Bhattaram et al.), and *in vitro* tests indicated that all the three minerals significantly reduced *V. cholerae* motility, adhesion to intestinal epithelial cells (Caco-2), and cholera toxin production (*ctxAB, fliA*, and *toxR*) *in vitro*, and decreased adhesion and toxin production in mouse intestine *ex vivo*. However, *in vivo* studies in an animal model are necessary to validate these results.

Bacterial pathogens persist in food processing facilities via growing predominantly as biofilms rather than in planktonic mode (Bae et al., 2012). Biofilms are complex microbial communities embedded in the protective extracellular polymeric substances (EPS). The efficiency of acidic electrolyzed water (AEW) to remove biofilms was evaluated for foodborne bacterial pathogens including *E. coli, V. parahaemolyticus*, and *L. monocytogenes*. *V. parahaemolyticus* infections (i.e., vibriosis) are normally acquired through exposure to sea water or through consumption of raw or undercooked contaminated seafood (Baker-Austin et al., 2018), while *L. monocytogenes* (a pathogen that can grow at refrigeration temperatures) infections (i.e., listeriosis) are frequently associated with unpasteurized dairy products and various ready-to-eat food (Fung et al., 2018). *In vitro* experiments showed that AEW triggered EPS disruption by the deformation of carbohydrate C-O-C bond and aromatic rings in amino acids tyrosine and phenylalanine. The authors suggested that AEW could be an eco-friendly alternative to sanitizers traditionally used in the food industry. Additionally, differential survival of hyper-aerotolerant *C. jejuni* under different gas conditions was also studied, and the resulting data suggested that modified atmosphere packaging using CO_2_ may help to controlling poultry contamination with hyper-aerotolerant *C. jejuni* (Oh et al.).

In summary, this Frontiers Research Topic includes 21 articles, and 146 authors from Austria, Canada, China, Denmark, Germany, Ireland, Japan, Portugal, South Korea, United Kingdom, and United States. It provides an overview of recent discoveries in resurgence, pathogenesis, and control strategies of the human serious foodborne pathogenic bacteria, and supports the urgent need for improving food safety and public health, particularly in globalization background. The information presented in the articles not only underscores future research areas and needs for scientists, but also benefits governments, food producers, food suppliers, and food consumers to work together toward eliminating and controlling pathogen persistence in food and resistant infections in humans.

## Author contributions

LC drafted the editorial. WA contributed to the editorial revision. All authors approved the final paper for publication.

### Conflict of interest statement

The authors declare that the research was conducted in the absence of any commercial or financial relationships that could be construed as a potential conflict of interest.
